# Anti-tumor activity of a novel proteasome inhibitor D395 against multiple myeloma and its lower cardiotoxicity compared with carfilzomib

**DOI:** 10.1038/s41419-021-03701-z

**Published:** 2021-04-30

**Authors:** Xuxing Shen, Chao Wu, Meng Lei, Qing Yan, Haoyang Zhang, Lina Zhang, Xueyuan Wang, Ye Yang, Jianyong Li, Yongqiang Zhu, Lijuan Chen

**Affiliations:** 1 Department of Hematology, the First Affiliated Hospital of Nanjing Medical University, Jiangsu Province Hospital, Collaborative Innovation Center for Cancer Personalized Medicine, Nanjing, 210029 China; 2 College of Science, Nanjing Forestry University, Nanjing, 210037 China; 3 College of life Science, Nanjing Normal University, Nanjing, 210046 China; 4 School of Medicine & Holistic integrative Medicine, Nanjing University of Chinese Medicine, Nanjing, 210023 China

**Keywords:** Myeloma, Drug development

## Abstract

Carfilzomib, a second-generation proteasome inhibitor, has significantly improved the survival rate of multiple myeloma (MM) patients, but its clinical application is still restricted by drug resistance and cardiotoxicity. Here, we identified a novel proteasome inhibitor, D395, and assessed its efficacy in treating MM as well as its cardiotoxicity at the preclinical level. The activities of purified and intracellular proteasomes were measured to determine the effect of D395 on the proteasome. CCK-8 and flow cytometry experiments were designed to evaluate the effects of D395 on cell growth and apoptosis. The effects of D395 and carfilzomib on serum enzyme activity, echocardiography features, cardiomyocyte morphology, and hERG channels were also compared. In our study, D395 was highly cytotoxic to MM cell lines and primary MM cells but not normal cells, and it was well tolerated in vivo. Similar to carfilzomib, D395 inhibited osteoclast differentiation in a dose-dependent manner. In particular, D395 exhibited lower cardiotoxicity than carfilzomib in all experiments. In conclusion, D395 is a novel irreversible proteasome inhibitor that has remarkable anti-MM activity and mild cardiotoxicity in vitro and in vivo.

## Introduction

Multiple myeloma (MM), which is characterized by clonal proliferation of plasma cells in the bone marrow (BM), constitutes more than 10% of hematological malignancies^1,2^. Historically, patients with MM usually had poor outcomes. The advent of proteasome inhibitors (PIs) has significantly improved the progression-free survival (PFS) and overall survival (OS) of patients with MM^3^. The proteasome degrades misassembled and misfolded proteins as well as short-lived elements such as signaling peptides that control vital activities, thereby playing important roles in the cell cycle, stress response, DNA repair, and antigen presentation^4^. PIs inhibit proteasome inactivity, leading to the accumulation of these substrate proteins and subsequent cellular dysfunction^5,6^. Despite the significant therapeutic benefits of PIs in MM patients, resistance to PIs seems to be inevitable, ultimately resulting in relapses^7,8^. In addition, side effects, such as neurotoxicity and cardiotoxicity, have greatly limited the clinical application of PI therapy. Thus, more effective proteasome inhibitors with fewer toxicities are needed to improve therapeutic efficacy and prevent tolerance in patients with MM.

Carfilzomib is a second-generation PI approved for patients with relapsed/refractory multiple myeloma (RRMM)^9^. In clinical practice, carfilzomib is routinely combined with dexamethasone (Kd) or lenalidomide and dexamethasone (KRd) to treat RRMM. A previous study by Dimopoulos MA et al. compared Kd versus bortezomib plus dexamethasone (Vd) and reported longer PFS and OS in the Kd group, and the use of Kd was also associated with a lower risk of painful peripheral neuropathy^10^. Another study also demonstrated that the KRd regimen was superior to the Rd (lenalidomide and dexamethasone) regimen in improving PFS and OS in RRMM^11^. The above studies established the effect of carfilzomib in patients with RRMM.

All of the approved PIs are associated with cardiotoxicity in the treatment of MM^12^. Among all the approved PIs, carfilzomib is most strongly associated with the occurrence of cardiotoxicity, with an incidence of 5–10% in carfilzomib-treated MM patients^13,14^. Additionally, the risk of cardiotoxicity remains even after the discontinuation of carfilzomib. Several clinical factors, including the concomitant use of immunomodulatory drugs, have been shown to be predictive of the occurrence of cardiotoxicity in MM patients treated with carfilzomib^14^. However, it is still very challenging to use these predictors to guide the use of carfilzomib, and the occurrence of symptomatic carfilzomib-induced cardiotoxicity remains inevitable.

D395 is a newly discovered epoxomicin proteasome inhibitor that is structurally distinct from carfilzomib. D395 has higher aqueous solubility and lower cardiotoxicity than carfilzomib. In the present study, we evaluated the proteasome inhibitory activity of D395 at the enzyme level and then in myeloma cells. We also examined the anti-MM activities of D395 in MM cell lines and MM patient-derived CD138^+^ cells as well as animal models. Since carfilzomib has been reported to be an osteoclast suppressor in multiple myeloma bone environments^15^, we also explored the effect of D395 on osteoclast differentiation. The cardiotoxicity of D395 was also assessed in this study.

## Materials and Methods

### Cell Culture

Human MM.1S (dexamethasone-sensitive), MM.1R (dexamethasone-resistant), ARP1, and RPMI-8226 MM cell lines were obtained from American Type Culture Collection (ATCC, Rockville, USA) and cultured in RPMI-1640 medium (Gibco-BRL, USA) supplemented with 10% fetal bovine serum (Gibco, USA) and 1% penicillin/streptomycin (Gibco, USA). All the cells are maintained in 5% CO_2_ at 37 °C and have been tested for mycoplasma contamination. Peripheral blood mononuclear cells (PBMCs) were collected from healthy volunteers. BM mononuclear cells and PBMCs from MM patients were isolated by Ficoll-Hypaque density-gradient separation^16^. CD138^+^ cells were isolated from BM mononuclear cells of MM patients using CD138^+^ Micro Beads and the Auto MACS magnetic cell sorter (Miltenyi Biotech, Germany). All primary samples were obtained with informed consent from patients with MM or healthy donors according to the Declaration of Helsinki. This study was approved by the Review Committee of the First Affiliated Hospital of Nanjing Medical University.

### Purified proteasome and non-proteasome assays

Proteasome chymotrypsin-like, caspase-like, and trypsin-like activities were determined using purified human erythrocyte-derived constitutive 20S proteasomes (cCP) and 20S immunoproteasome (iCP) (Boston Biochem, USA) as previously described^17,18^. A standard substrate-based assay of non-proteasome was performed as described (GenScript, China)^19^.

### Proteasome activity assays in vitro and in vivo

In vitro, the 20S proteasome activity of cells was detected by using the Proteasome-Glo Assay (Promega, USA) either after drug treatment for 4, 24, or 48 h in fresh medium. In vivo, blood samples from mice treated with drugs by intravenous injection were collected 2 h after injection to analyze 20S proteasome activity^20,21^.

### Cell viability, cell cycle, and apoptosis assay

Cell viability was detected by Cell Counting Kit-8 (CCK-8) (Dojindo Laboratories, Japan). MM cells were seeded on 96-well plates and placed in an incubator for 24 h. After being treated with drugs for another 72 h, cell viability was examined using the CCK-8 reagent. The CellTiter-Glo Luminescent Cell Viability Assay Kit (Promega, USA) was used to determine the viability of CD138^+^ cells and PBMCs. For flow cytometry, MM cells were seeded in 6-well plates and placed in an incubator for 24 h. Then, the MM cells were incubated with drugs for another 24 h and collected for flow cytometry analysis. The cell cycle was examined by flow cytometry using a propidium iodide (PI) staining assay kit (BD Biosciences, USA). Cell apoptosis was detected by flow cytometry using the Annexin V/PI Kit (BD Biosciences, USA).

### Western blot analysis

MM cells were collected for lysis in RIPA buffer (Beyotime, Shanghai) with protease inhibitor cocktail (Roche, Switzerland). Lysate protein samples were resolved by 10% SDS-PAGE, transferred to PVDF membranes (Millipore, USA), and probed with specific antibodies (CST, USA). Immunoreactive bands were revealed by a peroxidase-conjugated secondary antibody (CST, USA) followed by enhanced chemiluminescence (ECL) reagents (Bioworld, China). The following antibodies were used: anti-P53 antibody (cat:48818, CST, USA), anti-cleaved caspase3 antibody (cat:9661, CST, USA), anti-cleaved caspase9 antibody (cat:20750, CST, USA), anti-P21 antibody (cat:10355-1-AP, Proteintech, China), anti-ubiquitination antibody (cat:10201-2-AP, Proteintech, China), anti-GAPDH antibody (cat:60004-1-lg, Proteintech, China), and secondary antibodies (Vazyme Biotech, China).

### Quantitative real-time reverse transcriptase-polymerase chain reaction (qRT-PCR)

MM cells incubated with drugs were collected, and total RNA was isolated by TRIzol reagent (Thermo Scientific, USA). Quantitative real-time PCR was performed using PrimeScript RT Master Mix (Takara, Japan) and Hieff® qPCR SYBR Green Master Mix (Yeasen, China) according to the manufacturers’ protocols. The 2^−ΔΔCT^ method was used to calculate target gene mRNA levels. The primers are shown in Supplementary Table 1.

### NF-κB activity assay

The plasmids pcDNA3.1-(+)-NF-κB and pcDNA3.1-(+)-Renilla were mixed with Lipofectamine 2000 (Invitrogen, USA) and then transfected into HEK293T cells (Institute of Biochemistry and Cell Biology, China). The NF-κB/luciferase-transfected HEK293T cells were treated with carfilzomib or D395 for 6 h and then stimulated with TNF-α for 18 h. The activity of NF-κB was examined by the Dual-Luciferase® Reporter Assay System (Promega, USA) as previously described^22^.

### Pharmacodynamic assay

To detect 20S proteasome activity in vivo, KM/ICR mice (male, 12 weeks old) purchased from Nanjing University Model Animal Research Center were treated with carfilzomib (i.v., 5 mg/kg) or D395 (i.v., 5 mg/kg) by intravenous injection. After 2 h, the mice were sacrificed to collect blood, heart, liver, spleen, lung, and brain. The tissue samples were lysed by a tissue grinder, and the tissue suspension and the isolated serum were collected to test the activity of the 20S proteasome with Proteasome-Glo assay reagents as described above^21^.

### Tumor formation experiments in BALB/c nude mouse models

BALB/c nude mice (male, 4–6 weeks old) were purchased from Ziyuan Co., Ltd. (Hangzhou, China). ARP1 cells (1 × 10^6^ cells in 100 μl PBS) were injected subcutaneously into the hindquarters of each mouse. After 4 days, fifteen mice were randomly separated into three groups (control group: PBS, i.v. 100 μl/mouse; carfilzomib group: carfilzomib, i.v. 4 mg/kg; D395 group: D395, i.v. 4 mg/kg) for tumor therapy. All mice were observed every 2 days for at least 7 weeks. Tumor growth was assessed by calculating volume according to the following equation: volume = *a* × *b*^2^/2, where *a* is the widest length and *b* is the perpendicular diameter. On the appointed day, fifteen tumor tissue samples were removed from the mice for hematoxylin and eosin (H&E) staining and stained with Ki67 and CD138 antibodies. Investigators were blinded to the group allocation during measuring tumor size, weight, and performing staining. All animal procedures were approved by the Institutional Review Board of the College of Life Science, Nanjing Normal University (Approval No. IACUC-20200506).

### Electrophysiological assay

The pcDNA3.1-(+)-hERG plasmids (University of California, USA) were transfected into HEK293T cells (Institute of Biochemistry and Cell Biology, China). The activation of the hERG pathway was detected by the whole-cell patch-clamp technique at room temperature (22 ± 1 °C) using an AXON 200B amplifier through a digitizer (DigiData 1440A, Molecular Devices, USA) controlled by pClamp software (v10.0) as previously described^23^.

### Echocardiography assay, biochemical assay, and histopathological assay

Twenty-one C57BL/6 mice (male, 12 weeks old) were randomly assigned to different groups for intraperitoneal injection with vehicle (1% DMSO plus 5% 2-hydroxypropyl-β- cyclodextrin (HPβCD), carfilzomib (8 mg/kg) or D395 (8 mg/kg) on day 1, 3, 5, and 7. Left ventricular end-diastole diameter (LVEDD), left ventricular end-systole diameter (LVESD), left ventricular end-diastolic volume (LVEDV), left ventricular end-systolic volume (LVESV), fractional shortening (FS%), and ejection fraction (EF%) in all groups were examined at day 0 and day 7 by an ultrasound system (GE Healthcare, USA) with a 13-MHz linear transducer^24^. On day 7, the mice were sacrificed after an echocardiography test to harvest the serum for biochemical estimations. Biochemical estimations, including alanine aminotransferase (ALT), aspartate transaminase (AST), lactic dehydrogenase (LDH), creatine kinase-MB (CK-MB), and creatine kinase (CK), were tested using an autoanalyzer (Siemens, USA). Freshly harvested cardiac tissue was fixed in 4% buffered formalin solution for 24 h and embedded in paraffin wax. Sections (4–5 mm thick) were obtained from each sample and stained with H&E. Histologic observation was performed with a Nikon i55 optical microscope (Nikon Corporation, Japan).

### Osteoblast and osteoclast differentiation

Osteoblast differentiation was induced in MC3T3-E1 cells. The osteogenic differentiation medium contained 10 mM β-glycerophosphate, 100 nM dexamethasone, and 200 μM L-ascorbic acid in α-MEM supplemented with 10% fetal bovine serum and 1% penicillin/streptomycin. The osteogenic media were replaced twice per week with fresh media containing different concentrations of D395. Alizarin red S staining was performed after 21 days to quantify mineralization. For osteoclast development, Raw264.7 cells were seeded at a density of 15,000–30,000 per cm^2^ in a 24-well plate. Osteoclast differentiation was initiated by adding RANKL (15 ng/ml, R&D Systems, USA). The medium was refreshed every 2 days. Different concentrations of D395 were added with every medium change. TRAP staining was performed on day 5, and osteoclasts were identified as multinucleated (≥3). A tartrate-resistant acid phosphatase assay kit (Beyotime, China) was used to detect the TRAP activity of the supernatant of the osteoclast cells.

### Statistical analysis

Values represent the mean ± standard deviation (SD) values for at least two independent experiments. Two-tailed Student’s *t* test and one-way ANOVA were used for comparisons of two or more than two groups, respectively. Statistical significance was achieved when *P* < 0.05. Statistical analysis was performed using GraphPad Prism 5 (GraphPad Software, USA). All the experiments were replicated at least three times.

## Results

### D395 selectively inhibits proteasome chymotrypsin-like activity

The constitutive proteasome (cCP) and immunoproteasome (iCP) are two forms of the 20S proteasome containing three catalytic centers: caspase-like (C-L), trypsin-like (T-L), and chymotrypsin-like (CT-L). To confirm the inhibitory effects of D395 at these three catalytic sites, we performed a fluorescence substrate experiment with purified c20S and i20S proteasomes. Similar to carfilzomib, D395 was potent and highly effective in inhibiting the C-L, T-L, and CT-L activities of the proteasome (Table 1). Moreover, the inhibitory effect of D395 (IC_50_ = 1.7 nmol/L) on CT-L activity on iCP was larger than that of carfilzomib (IC_50_ = 8.1 nmol/L). Bortezomib (PS-341, Velcade), the first FDA-approved proteasome inhibitor licensed for myeloma, is associated with peripheral neurotoxicity in humans due to its inhibition of non-proteasome function. Hence, we also tested the inhibitory effect of carfilzomib and D395 on six non-proteasomes. As shown in Supplementary Fig. 1A, neither carfilzomib nor D395 exerted an inhibitory effect, which indicates that neither causes peripheral neurotoxicity in vitro.

**Table 1:** The enzyme biochemical activity profiles of D395 against the 20S proteasome.

	Proteasome activity IC_50_ (nmol/L) 1 h treatment					
	Chymotrypsin-like		Caspase-like		Trypsin-like	
Proteasome Inhibitor	20S	Immuno 20S	20S	Immuno 20S	20S	Immuno 20
D395	21.8 ± 7.5	1.7 ± 0.1	>2000	1990 ± 338	2175.5 ± 196.5	321.2 ± 23.8
Carfilzomib	11.6 ± 0.9	8.1 ± 0.5	>2000	1324.5 ± 71.5	2400.5 ± 181.5	432.7 ± 13.5

The enzyme activity of purified c20S and i20S proteasomes treated with carfilzomib and D395 for 1 h was detected by a fluorescence substrate experiment. Data are expressed as the mean ± SD.

### D395 inhibits cellular proteasome activity

**Fig. 1:**
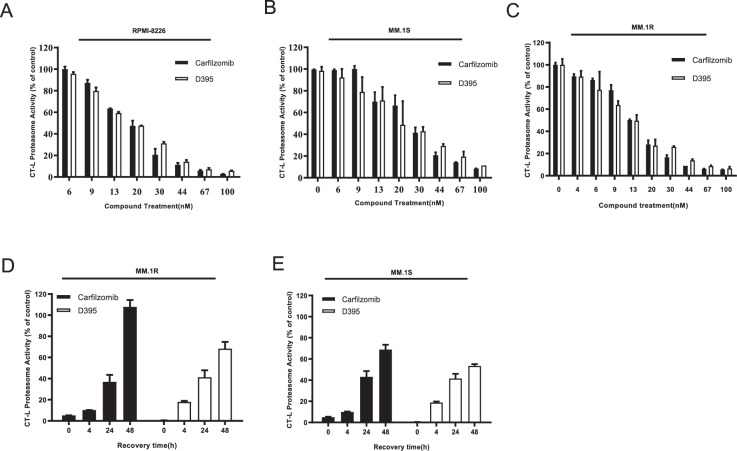
Inhibition and recovery of proteasome activity in MM cell lines. **A**–**C** Inhibition of cellular proteasome activity following D395 and carfilzomib treatment for 24 h. **D**, **E** Recovery of cellular proteasome activity following D395 and carfilzomib treatment. Proteasome chymotrypsin-like activity was measured in lysates prepared from RPMI-8226, MM.1S and MM.1R cells at the indicated time following exposure to various concentrations of D395 or carfilzomib.

To evaluate the ability of D395 to inhibit the CT-L activity of the proteasome in MM cell lines, MM.1S, MM.1R, and RPMI-8226 cells were treated with various concentrations of carfilzomib or D395 for 1 h. Proteasome activity was measured by the Proteasome-Glo cell-based assay. Dose-dependent proteasome activity decreased in all tested cell lines. The IC_50_ values of D395 were 23.4 nM, 13.2 nM, and 18.3 nM, and the IC_50_ values of carfilzomib were 25.8 nM, 13.8 nM, and 18.1 nM in RPMI-8226 cells (Fig. 1A), MM.1 S cells (Fig. 1B), and MM.1 R cells (Fig. 1C), respectively. In contrast to the bortezomib-proteasome complex, the carfilzomib-proteasome complex was irreversible due to its hydrolytic stability. To assess the recovery of proteasome activity in cells, CT-L activity in MM.1S (Fig. 1D) and MM.1R (Fig. 1E) cells was examined after drug treatment for 48 h. The recovery of proteasome activity in D395-treated cells was slower than that in carfilzomib-treated cells. These results suggest that D395 has a stronger inhibitory effect on proteasomes.

### D395 decreases the viability of MM cell lines and MM patient-derived BM CD138^+^ cells

**Fig. 2:**
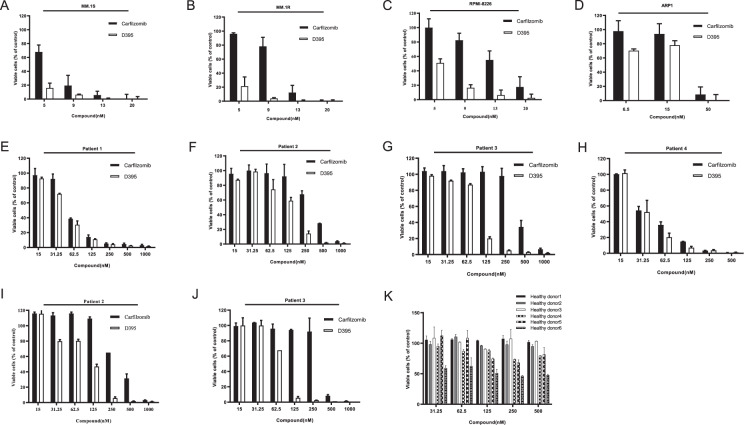
The cytotoxicity of D395 in MM cells. Four MM cell lines, MM.1S (**A**), MM.1R (**B**), RPMI-8226 (**C**) and ARP1 (**D**), were exposed to D395 or carfilzomib for 24 h followed by the cell cytotoxicity test. The CD138^+^ population isolated from three newly diagnosed MM patients (**E**–**G**) and one relapsed MM patient (**H**) were treated with different concentrations of D395 or carfilzomib for 24 h for the cell cytotoxicity test. **I**, **J** Patient 2- and patient 3-derived CD138^+^ populations were treated with various concentrations of D395 or carfilzomib for 36 h followed by CCK-8 test. **K** PBMCs from six healthy donors were treated with D395 or carfilzomib to evaluate cell cytotoxicity in normal cells.

To determine the cytotoxic effects of carfilzomib and D395 in vitro, human MM cell lines (MM.1S, MM.1R, RPMI-8226, and ARP1) were exposed to different concentrations of D395 or carfilzomib for 72 h. The Cell Counting Kit-8 (CCK-8) assay was used for cell viability assessment. CCK-8 provided a highly water-soluble tetrazole salt, WST-8, which would produce a water-soluble orange formazan in the presence of dehydrogenase derived from living cells. The OD value was detected at 450 nm with a microplate analyzer to indirectly reflect the number of living cells. A dose-dependent decrease in cell viability was noted in all four cell lines (Fig. 2A–D). In addition, CD138^+^ cells were purified from bone marrow aspirates of four MM patients and treated with carfilzomib or D395 for 24 h. Among the four patients, patients 1 (Fig. 2E), 2 (Fig. 2F), and 3 (Fig. 2G) were newly diagnosed, and patient 4 (Fig. 2H) had experienced relapse after prior therapies. An obvious dose-dependent decrease was observed in the viability of MM cells derived from all four patients after exposure to the drugs. In particular, in patient 2 and patient 3, D395 exerted a more potent cytotoxic effect than carfilzomib, and this superior effect became more significant after 36 h of treatment (Fig. 2I, J). In addition, D395, even at a higher concentration, did not trigger a significant decrease in the viability of PBMCs obtained from healthy volunteers (Fig. 2K). The cytotoxicity profiles of D395 and carfilzomib in representative MM cell lines and primary MM cells are presented in Supplementary Table 2.

### D395 induces apoptosis and cell cycle arrest in MM cells

**Fig. 3:**
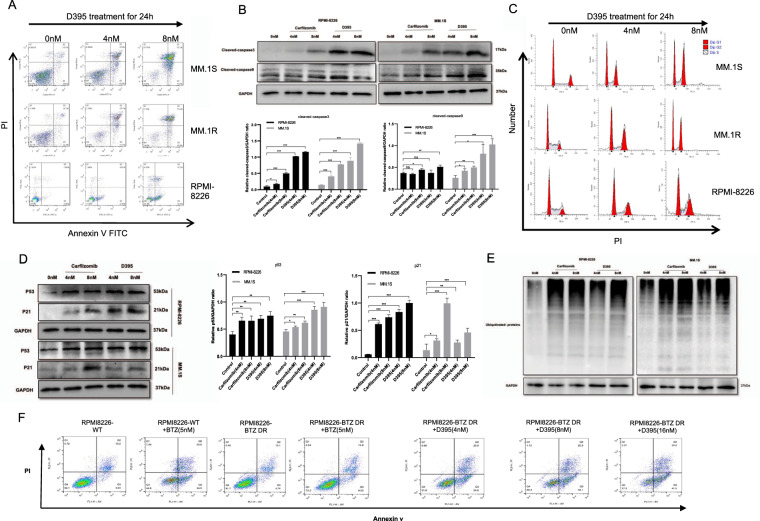
D395 induces MM cell apoptosis and inhibits the cell cycle. **A** The apoptotic population of MM cells following exposure to D395 were detected by cell surface annexin V staining followed by flow cytometry. **B** Apoptosis pathway-related markers, including cleaved caspase 3 and cleaved caspase 9, were detected in cell lysates from RPMI-8226 and MM.1S cells by western blot after drug treatment for 24 h. **C** The cell cycle distribution in RPMI-8226, MM.1S and MM.1R cells treated with D395 for 24 h was determined by flow cytometry analysis. RPMI-8226 and MM.1S cells were pretreated with D395 or carfilzomib for 24 h and then subsequently dissociated to determine the expression of the cell cycle‑associated proteins p53 and p21 (**D**) and polyubiquitinated proteins (**E**) by western blot. **F** RPMI-8226 WT and RPMI-8226 BTZ-DR cells were incubated with bortezomib (left) and different doses of D395 (right) then labeled with annexin V for flow cytometry.

To explore the mechanisms underlying the cytotoxic effects of D395 in MM cells, we performed cell cycle and apoptosis analyses. Flow cytometry indicated that D395 treatment increased the proportion of apoptotic cells (Fig. 3A). Cell apoptosis markers, including cleaved caspase3 and cleaved caspase9, were markedly upregulated in D395-treated cells (Fig. 3B). Cell cycle analysis revealed significant G2/M-phase arrest in MM cells after D395 treatment (Fig. 3C). Cell cycle-related proteins such as P53 and P21 were moderately upregulated in D395-treated cells (Fig. 3D). Obvious accumulation of polyubiquitinated proteins was found in cells treated with carfilzomib or D395 (Fig. 3E). Taken together, these results indicate that D395 exerts anti-tumor effects in MM by promoting cell apoptosis and inducing cell cycle arrest. It is known that drug resistance limits the application of proteasome inhibitors in clinical treatment. Therefore, the effect of D395 on the drug resistance cohort is important. As shown in Fig. 3F (left graph), the apoptotic population of the RPMI-8226 BTZ-DR group treated with bortezomib was lower than that of the RPMI-8226 WT group, indicating that we successfully constructed bortezomib-resistant cell lines. These bortezomib-resistant cells were next cultured with various concentrations of D395. Flow cytometry showed that D395-treated bortezomib-resistant cells exhibited a dose-dependent increase in cell death (Fig. 3F, right graph). Our results imply that D395 is cytotoxic to bortezomib-resistant cell lines.

### D395 inhibits the NF-κB signaling pathway

**Fig. 4:**
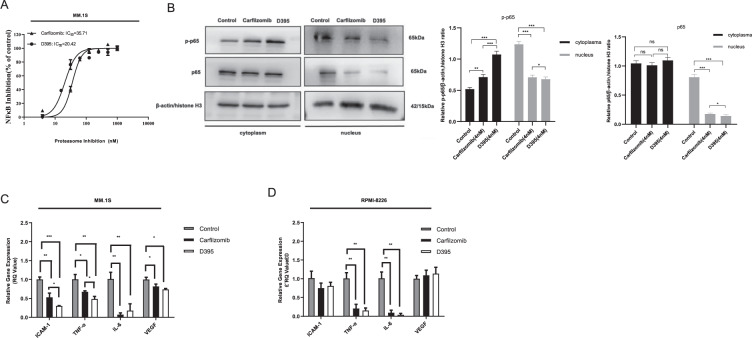
D395 modulates the NF-κB signaling pathway in MM cell lines. **A** HEK293T cells transfected with NF-κB/luciferase were incubated with increasing concentrations of D395 or carfilzomib for 6 h followed by 10 ng/ml TNF-α stimulation for another 18 h. Luciferase activity was determined by using the Dual-Luciferase Reporter Assay System. **B** RPMI-8226 cells were treated with D395 (4 nM) or carfilzomib (4 nM). After 24 h, cytoplasmic and nuclear proteins were isolated for western blot using anti-p-p65 and anti-p65 antibodies. **C**, **D** RPMI-8226 and MM.1S cells were treated with the indicated concentrations of D395 or carfilzomib for 24 h. Cell lysates were harvested, and total RNA was isolated. The expression of *ICAM-1, TNF-α, IL-6,* and *VEGF* was detected by qRT-PCR.

Proteasome inhibitors may function in MM therapy by blocking proteasomal degradation of IκBα to inhibit nuclear factor NF-κB activity^25^. Activation of NF-κB was determined by luciferase reporter gene assay using stably transfected NF-κB-luc HEK293T cells. The NF-κB signaling pathway was activated by TNF-α stimulation. D395 decreased luciferase activity more strongly than carfilzomib (IC_50_: 20.42 nM vs. 35. 71 nM, respectively; Fig. 4A). To better understand the mechanism of the effect of D395 on the NF-κB signaling pathway, related protein levels in RPMI-8226 cells were examined. As shown in Fig. 4B, p65 and p-p65 protein levels were substantially downregulated in the nucleus but were not changed in the cytoplasm, suggesting that D395 only exhibited its effect when the NF-κB signaling pathway was suppressed. Previous studies have demonstrated that crosstalk between MM cells and bone marrow stromal cells (BMSCs) leads to NF-κB activation and cytokine secretion, which promotes the survival and growth of MM cells. We found that the expression levels of cytokine genes (*ICAM1, TNF-α, IL-6, VEGF*) in MM.1S cells (Fig. 4C) and RPMI-8226 cells (Fig. 4D) were decreased after D395 treatment. However, only the expression of TNF-α and IL-6 was suppressed by D395 in RPMI-8226 cells. Since the RPMI-8226 cell line is a p53-deficient MM cell line and p53 plays an important role in tumor cell survival, this may be the explanation for these results^26,27^.

### D395 inhibits tumor growth in a xenograft mouse model

**Fig. 5:**
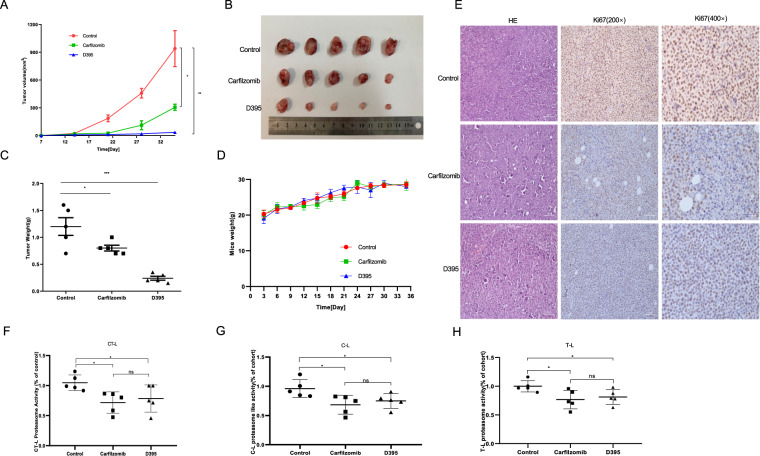
Effects of D395 on the growth of ARP1 xenografts established in nude mice. **A**–**C** ARP1 cells (1 × 10^6^ cells per mouse) were subcutaneously injected into BALB/c nude mice. After 4 days, the mice were randomly divided into 3 groups for different therapies (control group: PBS, i.v. 100 μl/mouse; carfilzomib group: carfilzomib, i.v. 4 mg/kg; D395 group: D395, i.v. 4 mg/kg). All the mice were sacrificed at day 35. Tumor volume and weight were recorded. **D** Mice body weight was measured every 3 days. Data are shown as the mean ± SD. **E** The obtained tumor samples were dissociated for immunohistochemistry and stained for Ki67 (scale bar=50 μm). **F**–**H** Proteasome CT-L, C-L, and T-L activities in tumors from control group, carfilzomib group and D395 group were detected.

We next validated the anti-tumor role of D395 in vivo. Human MM cell line ARP1 (1 × 10^6^ cells per mouse) were injected subcutaneously into BALB/c nude mice. Four days later, fifteen mice were randomly divided into three groups (control group: PBS, i.v. 100 μl/mouse; carfilzomib group: carfilzomib, i.v. 4 mg/kg; D395 group: D395, i.v. 4 mg/kg). The medication was given twice a week. Tumor size was measured every 3 days (Fig. 5A). Throughout the growth of the tumors, the tumor lumps in the carfilzomib and D395 treatment groups grew slower than those in the control group. In addition, D395 was superior to carfilzomib in restricting tumor growth. On the endpoint day, all the mice were sacrificed, and tumors were collected (Fig. 5B, C). During the treatment period, the weight of the mice did not change, suggesting that these two drugs had no impact on body growth (Fig. 5D). Tumor tissues were sampled for immunohistochemistry. The tissues were stained for Ki67, a cell proliferation marker, to show tumor growth (Fig. 5E). To better understand the role of D395 in tumor growth, we analyzed the proteasome activity in the tumor samples. Our results suggested that the CT-L, C-L, and T-L activities were lower in the carfilzomib and D395 groups than in the control group, although no significant difference was found between the carfilzomib and D395 groups (Fig. 5F–H). Thus, we concluded that carfilzomib and D395, acting as proteasome inhibitors, indeed inhibited tumor growth in vivo.

### D395 inhibits osteoclast differentiation

**Fig. 6:**
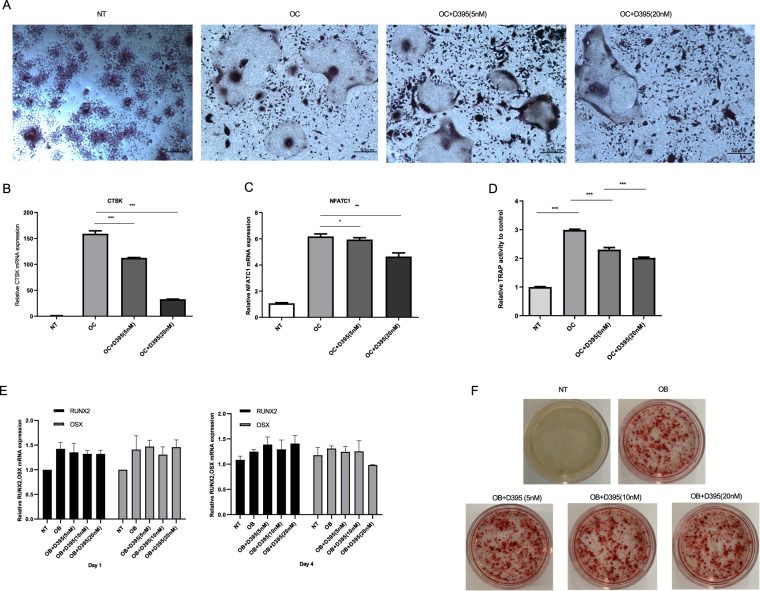
Effects of D395 on osteoclast and osteoblast differentiation. **A** Raw264.7 cells were differentiated into osteoclasts stimulated with RANKL (10 ng/ml) and different dose of D395 at the indicated time. TRAP staining was performed at day 5, and osteoclasts were characterized as multinucleated (≥3) (scale bar = 50 μm). **B**, **C** qRT-PCR was used to examine the expression of genes related to osteoclast formation, including *Ctsk* and *Nfatc1*. **D** TRAP activity in the supernatant of osteoclast cells was detected by Tartrate-Resistant Acid Phosphatase Assay Kit on day 5. **E** MC3T3-E1 cells cultured in osteogenic medium with different concentrations of D395 were used for osteoblast differentiation. The expression of genes related to osteogenesis was examined by qRT-PCR at day 1 and day 4. **F** Alizarin red S staining was performed on day 21 to assess osteoblast differentiation. (NT, no treatment group; OC, osteoclast formation positive control with RANKL (10 ng/ml) added.).

Carfilzomib inhibits pathological bone destruction in patients with MM. However, the effect of D395 on bone metabolism is unknown. Here, we used the osteoclast and osteoblast differentiation model to determine whether D395 affects bone resorption and formation. Raw264.7 cells were incubated with different concentrations of D395 as indicated. Osteoclast formation was examined by TRAP staining on day 5 (Fig. 6A). TRAP is a specific enzyme marker of osteoclasts. TRAP hydrolyze naphthol AS-BI phosphate into naphthol AS-BI in the presence of potassium sodium tartrate, which combines with chromogenic agent to form red precipitates. D395 significantly suppressed osteoclast differentiation, as shown by the presence of fewer mature osteoclasts. The osteoclast differentiation-related genes *Ctsk* and *Nnfatc1* were detected by qPCR on day 4 (Fig. 6B, C). Furthermore, the cell supernatant was collected for TRAP activity detection. The D395 group showed decreased TRAP activity compared to the control group (Fig. 6D). However, D395 failed to increase osteoblast formation in MC3T3-E1 cultures, and both osteoblast differentiation-related gene expression and Alizarin Red S staining showed negative results after D395 treatment (Fig. 6E, F).

### D395 is less cardiotoxic than carfilzomib in vivo

**Fig. 7:**
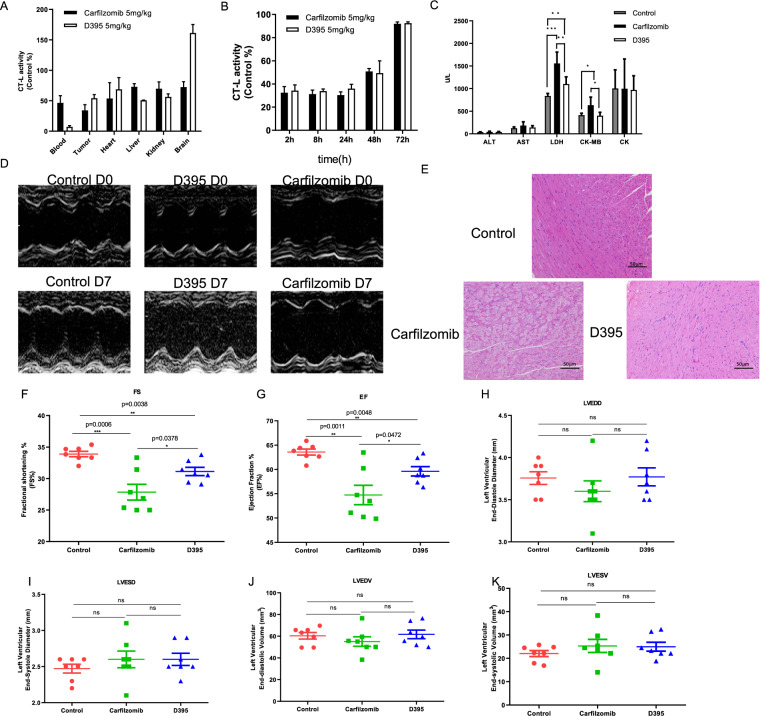
D395 is significantly less cardiotoxic than carfilzomib in vivo. Carfilzomib (5 mg/kg) or D395 (5 mg/kg) were injected into RPMI-8226 bearing mice for 1 h, respectively. **A** CT-L activity was detected in organs including blood, tumor, heart, liver, kidney, and brain. **B** The recovery of CT-L activity was evaluated in blood at 2 h, 8 h, 24 h, 48 h, and 72 h. **C** The levels of CK, CK-MB, LDH, ALT, and AST in the serum of carfilzomib and D395-treated mice were detected by an autoanalyzer. Data are expressed as the mean ± SD. **D** C57BL/6 mice were randomized to different treatment groups and administered vehicle (1% DMSO and 5% HPβCD), carfilzomib (8 mg/kg) or D395 (8 mg/kg) by intraperitoneal injection every 2 days for 1 week. Representative echocardiographic M-mode images of mice at baseline (day 0) and endpoint (day 7) are shown. **E** H&E staining showed morphological changes in heart histopathology in the different experimental groups (scale bar = 50 μm). **F** Echocardiographic assessment of cardiac function in mice was used to record FS, EF, LVEDD, LVESD, LVEDV, and LVESV (LVEDD: Left ventricular end-diastole diameter; LVESD: Left ventricular end-systole diameter; LVEDV: Left ventricular end-diastolic volume; LVESV: Left ventricular end-systolic volume; FS: Fractional shortening; EF: Ejection fraction; ALT: Alanine aminotransferase; AST: Aspartate transaminase; LDH: Lactic dehydrogenase; CK-MB: Creatine kinase-MB; CK: Creatine kinase).

We then assessed the cardiotoxicity of D395 in vivo. First, CT-L activities in the blood, tumor, heart, liver, kidney, and brain were detected at 1 h after D395 injection into RPMI-8226-bearing mice. As shown in Fig. 7A, except for blood and brain, the CT-L activity in other tissues was equally inhibited. In addition, CT-L activity recovered in a time-dependent manner, as previously discovered in vitro (Fig. 7B). Next, we evaluated the effect of D395 on cardiac function. Carfilzomib and D395 were intraperitoneally injected into C57BL/6 mice every 2 days for 1 week. The echocardiographic analysis was performed at baseline (day 0) and endpoint (day 7) (Fig. 7D). Compared to those in the control group, the FS% and EF% decreased in both the carfilzomib and D395 groups, but less notably in the D395 group (Fig. 7F, G). Importantly, the D395 group showed moderately higher FS% and EF% than the carfilzomib group (FS%: carfilzomib vs. D395, *p* = 0.0378; EF%: carfilzomib vs. D395, *p* = 0.0472). No significant differences in LVEDD, LVESD, LVEDV, or LVESV were found among the carfilzomib group, the D395 group, and the control group (Fig. 7H–K). Histological analysis demonstrated myocardial degeneration and myocardial fiber damage with cytoplasmic vacuoles in the carfilzomib group, and most of these changes were not observed in the D395 group (Fig. 7E). We also examined the levels of CK, CK-MB, LDH, ALT, and AST in the blood serum from the treated mice. CK-MB and LDH levels were increased in the carfilzomib group but remained low in the D395 group (Fig. 7C). In conclusion, D395 was less cardiotoxic than carfilzomib.

### Effects of D395 and carfilzomib on hERG channels

**Fig. 8:**
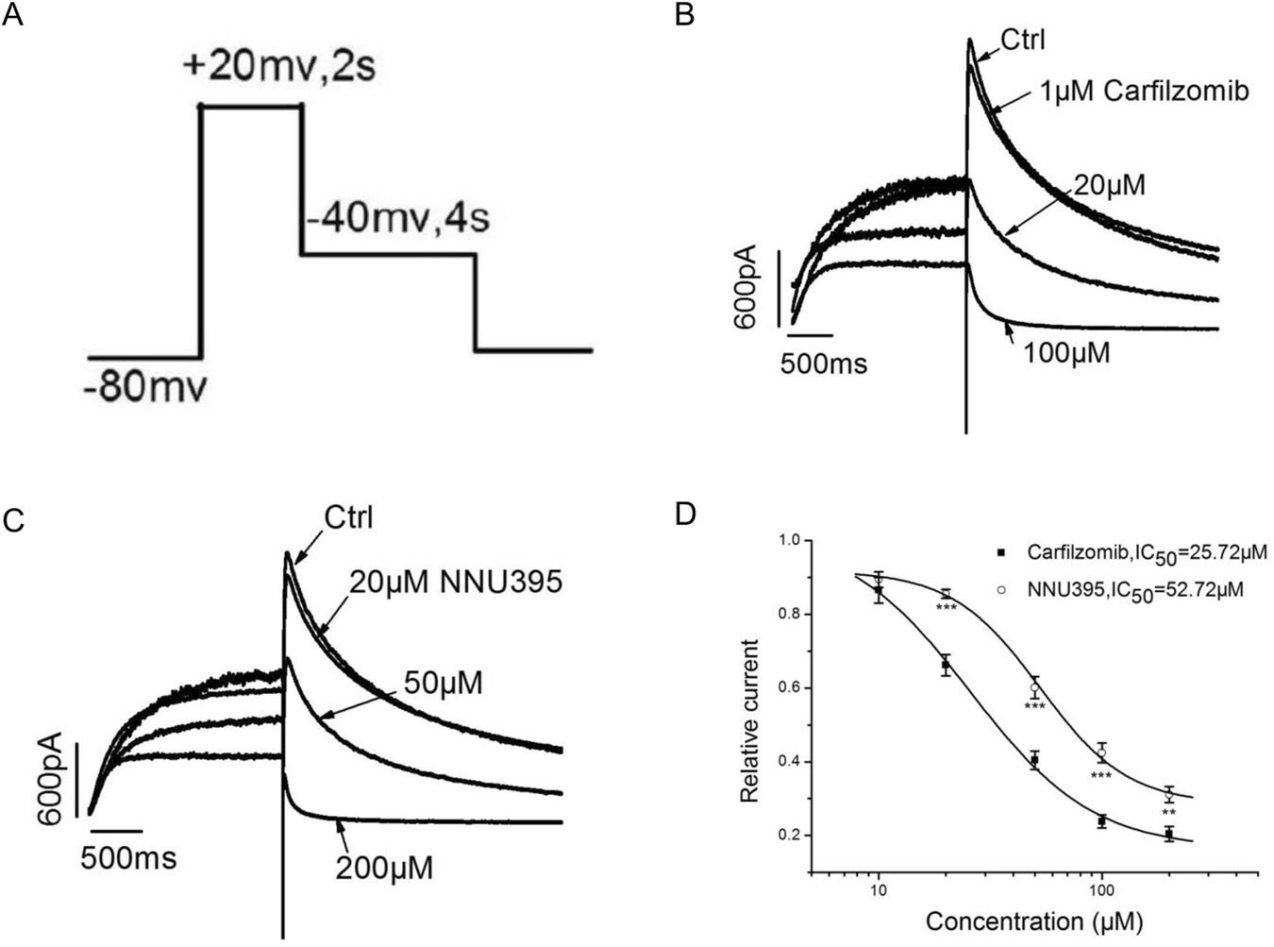
Effects of D395 and carfilzomib on hERG channels. **A** The command waveform of the tail-current protocol under voltage-clamp mode. **B**, **C** Increasing doses of D395 (20 µM, 50 µM, and 200 µM) or carfilzomib (1 µM, 20 µM, and 100 µM) inhibited outward potassium currents. **D** The inhibitory activities of D395 and carfilzomib in tail current were merged and shown as a diagram form.

Human ether-à-go-go-related gene (hERG) K^+^ channels are vital in cardiac repolarization^28,29^. Drug cardiotoxicity can be partially attributed to hERG channel dysfunction that leads to long QT syndrome and ventricular action potential delay. Thus, we compared the effects of carfilzomib and D395 on hERG channels. The real-time current was measured during drug treatment. Figure 8A presents the command waveform of the tail-current protocol under voltage-clamp mode. As shown in Fig. 8B, C, both carfilzomib and D395 significantly inhibited outward potassium currents in a dose-dependent manner compared with the control treatment. Remarkably, the cardiac toxicity characterized by outward K^+^ tail-current inhibition was lower in the D395 group than in the carfilzomib group (Fig. 8D, IC_50_: 52.72 μM vs. 25.72 μM), respectively.

## Discussion

Our study first showed the effects of D395 on the activity profiles of the ubiquitin-proteasome complex. We demonstrated that the CT-L activities of subunits β5c (c20s) and β5i (i20s) were inhibited by both D395 and carfilzomib, and the inhibitory effect was more pronounced with the D395 treatment. Similar results were observed in different MM cell lines. Although the binding of D395 to the proteasome is irreversible, the CT-L activity in MM cells treated with D395 recovered by more than 60% within 48 h, which could be attributed to the induction of mRNA transcription and de novo synthesis of subunits that could be added to new proteasomes^30,31^. We found that D395 had a more persistent effect on CT-L activity than carfilzomib. Notably, D395 showed a lower IC_50_ than carfilzomib, suggesting the stronger anti-MM activity of D395 through regulating cell apoptosis and cell survival. In addition, D395 demonstrated no toxicity in PBMCs of healthy volunteers, suggesting that it could be specific and safe in clinical treatment. Finally, we confirmed the anti-MM activity of D395 in vivo using mouse models. In summary, both in vitro and in vivo studies indicated that D395 had stronger anti-MM activity than carfilzomib.

Osteoblasts (OBs) and osteoclasts (OCs) are involved in balancing bone formation and bone resorption. The hyperactivity of OCs and hypoactivity of OBs are responsible for MM bone disease. Bone destruction and subsequent hypercalcemia are common clinical conditions that can be relieved by some proteasome inhibitors and immunomodulators. In a previous study, carfilzomib demonstrated a strong inhibitory effect on bone resorption by attenuating the RANKL-mediated signaling pathway and blocking proteasomal degradation of HDAC4 in OBs and NF-kB activity in OCs^15^. In another study, a new Bruton’s tyrosine kinase (Btk) inhibitor, CC-292, combined with carfilzomib inhibited OC differentiation by downregulating the expression of c-Src, Pyk2, and cortactin^32^. Given the role of carfilzomib in bone remodeling, we then explored the effects of D395 on bone metabolism. Our data suggested that D395 strongly inhibited the differentiation of OCs from Raw264.7 cells, a typical mononuclear macrophage cell line for OC formation. However, D395 showed no effect on OBs formation.

PIs display various levels of cardiotoxicity. Between bortezomib and carfilzomib, the latter was associated with a higher rate of adverse cardiovascular effects than the former. These adverse cardiovascular effects could be related to the inhibition of the proteasome in the heart. An in vivo study by Efentakis et al. showed that carfilzomib damaged the myocardium by downregulating AMPKα and autophagy-related proteins^24^. This study also found that carfilzomib-induced transient vascular impairment and oxidative burst. In the current study, we provided evidence that D395 induced milder cardiotoxicity than carfilzomib. Mice cardiac ultrasound demonstrated that D395 induced smaller reductions in FS% and EF% than carfilzomib, which was consistent with the electrophysiological results. Moreover, serum LDH and CK-MB activities were significantly upregulated in the carfilzomib group compared with the D395 group. However, there were no significant differences in AST and CK activities between the two groups. We speculate that AST and CK might have increased to accelerate recovery from myocardial injury and then returned to normal levels before we collected the blood of the mice. In addition, H&E staining illustrated that D395 caused less damage to myocardial cells than carfilzomib. Blockade of the human ether-à-go-go-related gene (hERG) channel leads to fatal cardiotoxicity, which is the reason for the severe restrictions on the use of many approved drugs. In the present study, we compared the effects of carfilzomib and D395 on hERG channels and found that cardiotoxicity, as revealed by outward K^+^ tail current inhibition, was lower in the D395 group. Assessment of hERG channel blockade by using patch-clamp methodology in HEK 293 cells has been used to predict cardiotoxicity in fifty compounds^33^. In the study by Cai et al., a deep learning approach named deep hERG was developed to identify hERG channel blockers to predict drug-induced cardiotoxicity. All of these studies suggest an unmet need for agents with lower cardiotoxicities.

Taken together, the data shown in the present study demonstrated that the novel proteasome inhibitor D395 exerted stronger anti-MM activity than carfilzomib both in vitro and in vivo. Most importantly, D395 showed substantially fewer cardiotoxic effects than carfilzomib, suggesting its safety in clinical practice in the future.

## Supplementary information

supplemental figures and tables
