# Supplementary material for: Anti-tumor activity of a novel proteasome inhibitor D395 against multiple myeloma and its lower cardiotoxicity compared with carfilzomib
Source: Cell Death Dis. 2021 Apr 30;12(5):429. doi: 10.1038/s41419-021-03701-z (PMC8087809; doi:10.1038/s41419-021-03701-z)
Supplement: Supplementary file 1 — supplemental figures and tables [file 41419_2021_3701_MOESM1_ESM.docx]

**Table 1. Primers for the qRT-PCR.**

| **Name** | **Sequence** |
| --- | --- |
| ICAM1_F | TTGGGCATAGAGACCCCGTT |
| ICAM1_R | GCACATTGCTCAGTTCATACACC |
| TNF-α_F | GAGGCCAAGCCCTGGTATG |
| TNF-α_R | CGGGCCGATTGATCTCAGC |
| IL-6_F | ACTCACCTCTTCAGAACGAATTG |
| IL-6_R | CCATCTTTGGAAGGTTCAGGTTG |
| VEGF_F | AGGGCAGAATCATCACGAAGT |
| VEGF_R | AGGGTCTCGATTGGATGGCA |
| CTSK_F | AGCAGGCTGGAGGACTAAGGT |
| CTSK_R | GATTTGTGCATCTCAGTGGAAGAC |
| NFATC1_F | CCTTATGTGGCTCAGGTCTTACTTC |
| NFATC1_R | TGGTCCCCGAGACCACAAT |

**Table 2. Cytotoxicity profile of D395 and Carfilzomib in representative MM cell lines and primary MM cell.**

| Cell type | | Treat time | Cell viability  IC_50_(nmol/L) | |
| --- | --- | --- | --- | --- |
|  |  |  | Carfilzomib | D395 |
| MM cell line | MM.1S | 72h | 7.0±0.9 | 4.7±0.3 |
|  | MM.1R | 72h | 11.0±0.9 | 4.7±0.5 |
|  | RPMI-8226 | 72h | 13.5±1.9 | 5.3±1.1 |
|  | ARP1 | 72h | 35.6±5.1 | 21.9±0.7 |
| Primary MM cell | pantiet 1 | 24h | 56.5±2.5 | 46.2±2.8 |
|  | pantiet 2 | 24h | 354.1±19.4 | 126.2±21.8 |
|  | pantiet 2 | 36h | 339.3±22.8 | 108.5±6.2 |
|  | pantiet 3 | 24h | 444.3±48.0 | 93.3±2.3 |
|  | pantiet 3 | 36h | 287.6±19.5 | 61.0±0.1 |
|  | pantiet 4 | 24h | 38±5.8 | 35.7±7.0 |

**Figure 1 The inhibitory effect of Carfilzomib and D395 on six non-proteasomes.**


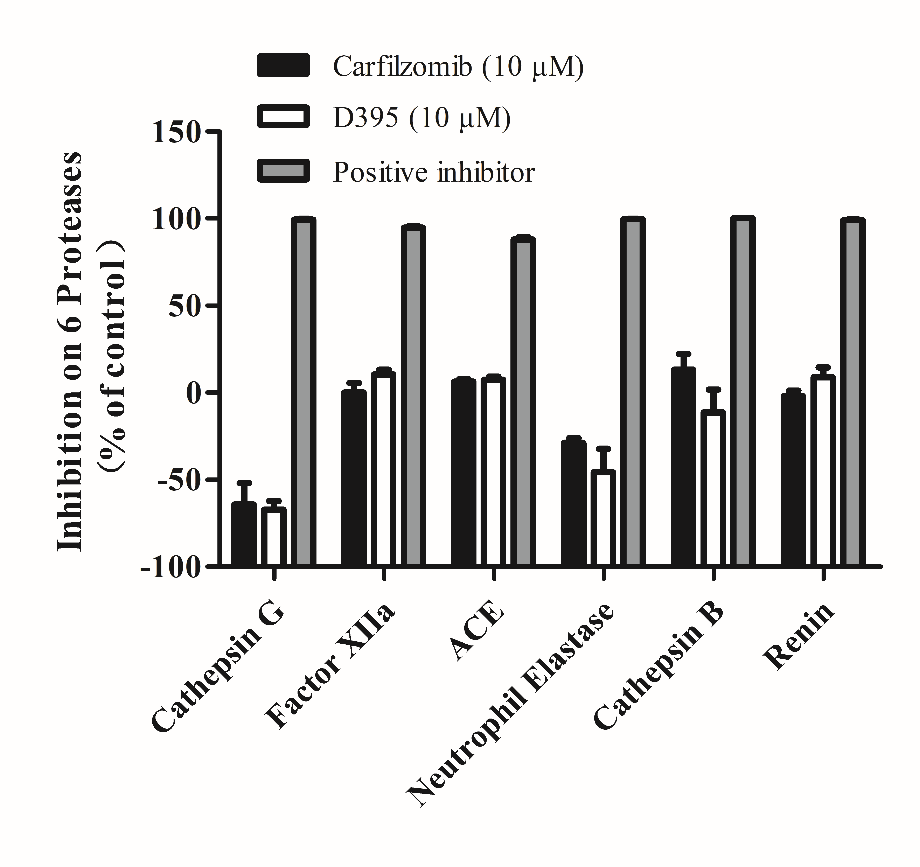


The inhibitory effect of carfilzomib and D395 on six non-proteasomes was detected by Proteasome-Glo Assay.
